# The Application of a Plant Biostimulant Based on Seaweed and Yeast Extract Improved Tomato Fruit Development and Quality

**DOI:** 10.3390/biom10121662

**Published:** 2020-12-12

**Authors:** Giuseppe Mannino, Cristina Campobenedetto, Ivano Vigliante, Valeria Contartese, Carla Gentile, Cinzia M. Bertea

**Affiliations:** 1 Department of Life Sciences and Systems Biology, Innovation Centre, Plant Physiology Unit, University of Turin, 10135 Turin, Italy; giuseppe.mannino@unito.it (G.M.); cristina.campobenedetto@unito.it (C.C.); 2 Green Has Italia S.p.A, 12043 Canale (CN), Italyi.vigliante@greenhasitalia.com (I.V.); v.contartese@greenhasitalia.it (V.C.); 3 Department of Biological, Chemical and Pharmaceutical Sciences and Technologies (STEBICEF), University of Palermo, 90128 Palermo, Italy; carla.gentile@unipa.it

**Keywords:** *Solanum lycopersicum*, polyphenols, lycopene, tocopherols, carotenoids, mineral content, fruit quality, ripening time, fruit size, fruit quality

## Abstract

Plant biostimulants are under investigation as innovative products to improve plant production and fruit quality, without resulting in environmental and food contaminations. Here, the effects of the application of Expando, a biostimulant based on seaweed and yeast extracts, on plant productivity, fruit ripening times, and fruit quality of *Solanum lycopersicum* var. Micro-Tom were evaluated. After biostimulant treatment, a two-week reduction of ripening times and a concomitant enhancement of the production percentage during the earliest ripening times, in terms of both fruit yield (+110%) and size (+85%), were observed. Concerning fruit quality, proximate analysis showed that tomatoes treated with the biostimulant had better nutritional composition compared to untreated samples, since both the quality of unsatured fatty acids (C16:3ω3: +328%; C18:2ω6: −23%) and micronutrients essential for human health (Fe: +14%; Cu: +21%; Zn: +24%) were increased. From a nutraceutical point of view, despite strong changes in bioactive compound profile not being observed, an increase of the antioxidant properties was recorded in fruits harvested by plants treated with the biostimulant (2,2’-azino-bis(3-ethylbenzothiazoline-6-sulphonic acid (ABTS): +38%; 2,2-diphenyl-1-picrylhydrazyl (DPPH): +11%). In conclusion, the biostimulant application was able to reduce the ripening times and fruit size, while slightly increasing nutritional and nutraceutical values, leading to more marketable tomato fruits.

## 1. Introduction

In the last decades, the world population has been constantly growing, and it is estimated to reach 10.6 billion in 2050 [1]. Feeding the current world population is considered already today a hard challenge. To date, waste reduction, changing diet habits, and enhancing the potential production of crops is applied as a strategy to monitor this global warning [2]. On the other hand, considering global climate changes, salinization, desertification, natural erosion, and fertility reduction of soils, finding new areas to use as agricultural land is not an easy task to achieve [1]. Consequently, the improvement of plant resilience to adverse conditions and the improvement of crop production by using agrochemicals and organic fertilizers has largely been employed in the agronomic practices [2,3]. However, in the last century, the use of these products massively increased worldwide, resulting not only in a well-awaited intensification of food production, but also in serious environmental contaminations [2,4]. In addition, scientific evidence suggests that the use of agrochemicals may affect crop quality, in terms of sensorial, nutritional, and nutraceutical traits [2,3,4], and is also a potential risk for human health [5,6].

Food safety is nowadays a primary concern for the vast majority of consumers [2,4], and in particular they are worried about chemical hazards, especially those perceived to be invisible and having long term effects or serious health implications [5,6]. For this reason, together with consumer awareness toward the benefits of food intake with high nutritional and nutraceutical properties [7,8], consumers’ preference for organic foods grown in an eco-sustainable way is also rising because they are perceived to be safe, healthy, and environmentally friendly [9].

In order to satisfy both agronomic and consumer needs, sustainable agriculture should be a valid alternative to conventional techniques, providing food sufficiently for all, while reducing environmental risks and allowing the production of high quality foods [10]. Moreover, changes in current unsustainable agricultural practices into more sustainable procedures may not only contribute to food security but also to mitigating climate change [11]. In this context, the use of plant wastes as natural biostimulants has aimed to increase sustainable productivity, and quality of fruit has turned out to be one of the most attractive frontiers in the agronomic field [12,13,14,15,16]. Despite both the differences in the raw materials employed and the large variety of phytochemicals present in the various biostimulant formulations [17], their application in very small amounts seems to be enough to promote plant growth. In particular, scientific evidence shows that treatment with biostimulants can affect plant growth processes by enhancing water and/or nutrient uptake [18], root and shoot growth [12,14,19], activity of key enzymes involved in primary or secondary metabolism [17,20], and plant antioxidant defense system [21]. Moreover, treatments with biostimulants seem to be able to positively affect fruit quality in terms of nutritional values and nutraceutical properties [13,14,15,16]. However, with the exception of few cases, the effects of biostimulant treatment on crops was mainly investigated at the seedling stage, or measuring plant physiological parameters under abiotic stresses [18,21,22,23]. Moreover, despite the literature presenting a few scientific papers relating to the effects derived from the application of commercial biostimulants [24,25], little knowledge is available on the effect of biostimulant application on fruit yield, proximate composition, and nutraceutical properties in the absence of environmental stress.

Here, we evaluated the potential effect derived from the application of a commercial biostimulant based on seaweed and selected yeast extracts in terms of productivity, pomological traits, proximate composition, and nutraceutical values of tomatoes (*Solanum lycopersicum* L. var. Micro-Tom) produced by plants grown in standard conditions. Seaweed and yeast extracts are receiving a greater acceptance in horticulture as plant biostimulants due to several beneficial effects against abiotic stresses [17,26,27,28]. However, the effects deriving from the application of biostimulants based on the combination of seaweed and yeast extracts on tomato fruit quality grown under standard conditions are scarcely available in the literature.

In this work, in order to understand if the biostimulant could affect plant productivity, fruit ripening times, and fruit quality, we monitored tomato plants after the application of the biostimulant under controlled conditions. In particular, concerning the evaluation of the tomato quality, we evaluated both nutritional (macro- and micro-nutrients) and nutraceutical (total polyphenol content, total carotenoid content, and antioxidant activities).

## 2. Materials and Methods

### 2.1. Plant Material and Treatments with the Biostimulant

Tomato (*Solanum lycopersicum* L. var. Micro-Tom) seeds were sowed in greenhouse under controlled temperature (day: 25 ± 3 °C; night: 18 ± 4 °C) and humidity (63% ± 6%) conditions. At the first true leaf appearance, tomato plants were transplanted in pots containing soil (50% unfertilized peat + 50% expanded clay) and grown until fruit production stage. For the experimentation, treatments were performed using a commercial biostimulant (Expando^®^, Green Has Italia S.P.A., Canale, Italy) applied via foliar application. The label of the product claims to contain 3% (*w*/*w*) organic nitrogen, 4% (*w*/*w*) phosphoric anhydride, 6% (*w*/*w*) potassium oxide, 0.02% (*w*/*w*) boron, 0.1% (*w*/*w*) molybdenum, 0.02% (*w*/*w*) manganese, and 12% (*w*/*w*) organic carbon. The pH (in 1% (*w*/*w*) water solution) and electrical conductivity (in water solution 1g L^−1^) were respectively 6.50 ± 0.50 and 350 µS cm^−1^. Concerning the treatments, the plants were divided into 3 different groups. The first one included 20 plants that were treated 3 times (during flowering, fruit-set, and fruit growing stages) with a biostimulant dosage equal to 1.5 mL/L^−1^ (triple treatment). The second groups included plants that received an additional biostimulant application during ripening phase at the same dosage (quadruple treatment). The last group was composed by plants treated using the same experimental protocol but with replacing the biostimulant with water. All plants received the same maintenance, and Hoagland solution was applied to the plants twice a week. Treatment application was performed using a hand sprayer until the complete wetting of all foliar surfaces (run-off condition), and without the use of surfactants.

### 2.2. Evaluation of Productivity and Pomological Analysis

**Figure 1:**
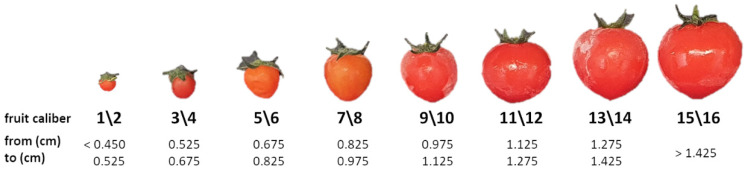
Tomato caliber classes and their relative external diameter (cm). After the last harvesting time, all fully colored fruits were grouped and photographed. In order to divide the fruits into the different size classes, we analyzed the resulting images by ImageJ.

Sixty days after the sowing, tomato fruits were harvested upon reaching a full and uniform red color. Fruits that met ripening criteria were collected every 4 days over a 30-day period. For each harvest time (HT), the fruits were counted and weighted using a digital scale (Cubis Sartorius, Gottingen, Germany). Yield per plant was determined by collecting all fruits from each plant. Moreover, after the last HT, the fruits of each tomato plant were grouped and photographed using a digital camera (Canon EOS M100, Tokyo, Japan). The resulting images were analyzed using ImageJ software and the external diameter (ED) was automatically calculated. On the basis of this parameter, we divided the tomatoes into 16 different fruit size classes, as shown in Figure 1.

### 2.3. Proximate Composition

#### 2.3.1. Sugar Content

The total content of sugars was determined as previously described [8]. Briefly, the fresh tomatoes were pulped and homogenized, and the resulting juices were vortexed (4000× *g*, 4 °C). Two grams of the centrifuged juice were mixed with 1 mL of 5% (*v*/*v*) phenol (Sigma Aldrich, Waltham, MA, USA) in a glass tube, and subsequently 5 mL of H_2_SO_4_ was rapidly added to the mixture. During the 10 min of incubation time, carbohydrates contained in the juice produced furfural derivatives that were spectrophotometrically detected at 490 nm. Reference solutions were prepared in the same manner but replacing the juice with distilled water. The measure was repeated twice. Quantification was performed using an external calibration curve of glucose, and the results were expressed as milligram per 100 g of fresh weight (FW).

#### 2.3.2. Protein Content

In order to quantify the protein content in tomatoes, we employed the Kjeldahl method as previously described [8,29]. Briefly, 1 gram of dried tomato sample was accurately weighed and then digested at 420 °C for 1 h with 10 mL of H_2_SO_4_. Then, 15 mL of 30% (*w*/*v*) H_2_O_2_ was slowly added in the presence of 0.2 g of CuO (8S Heating Digester, Velp Scientifica, Usmate Velate, Italy). Digested samples were then distilled using UDK Semi-Automatic Kjeldahl Distillation Unit (Velp Scientifica, Usmate Velate, Italy). In order to produce an alkaline distillation environment, we employed 32% (*w*/*v*) NaOH and distilled ammonia. Finally, each sample was separately collected in a volumetric flask containing 25 mL of 4% (*w*/*v*) H_3_BO_3_. The titrations were performed with standardized 0.25 N HCl. The total protein content was indirectly calculated using a nitrogen conversion factor of 6.25 [30].

#### 2.3.3. Lipid Content and Fatty Acid Profile

Fifty grams of dried tomato was refluxed in a Soxhlet apparatus using 250 mL of petroleum benzene in a weighed glass flask, as previously described [31]. The oils were recovered by distilling the organic solvent in a rotary evaporator at 50 °C and until a constant weight was measured. Fatty acid profile was obtained via trans-esterification of 500 mg of tomato sample with 1 mL of 10% (*w*/*v*) boron tri-fluoride solubilized in methanol [31,32]. Fifty micrograms of heptadecanoic acid (C17:0) were added in each sample as internal standard. The obtained fatty acid methyl esters (FAMEs) were purified by the serial addition of 1 mL water and 1 mL hexane. Purification was repeated twice, and the organic phases were pooled together. After centrifugation (8000× *g*, 5 min, 4 °C) and dehydration by using anhydrous MgSO_4_, the phases containing FAMEs were employed for gas chromatographic (GC) analysis. FAME identification was performed by Gas Chromatography (GC) coupled with Mass Spectrometry (MS) (GCMS-TQ8040, Shimadzu, Kyoto, Japan) and quantified by Gas Chromatography with Flame Ionization Detection (FID) (GC-2010 Plus, Shimadzu, Kyoto, Japan). For the quantification, an external curve of heptadecanoic-methyl ester (Limit of Detection: 0.2 μg/mL; Limit of Quantification: 0.7 μg/mL) was employed. Helium was used as GC carrier during the analysis with a constant flux of 1 mL min^−1^. The chromatographic separation was obtained in ZB5-MS (30 m length, 250 μm diameter and stationary phase thickness of 0.25 μm, 5% phenyl-arylene, and 95% poly-dimethyl siloxane) column (Phenomenex, Torrance, CA, USA). Chromatographic temperature conditions employed for the separation consisted of 60 °C held for 1 min, and then raised 10 °C per minute until 180 °C. Temperature was then brought to 230 °C in 40 min and to 320 °C in 20 min. This temperature was held for an additional 5 min. The same column and chromatographic conditions were used for both GC–MS analyses. Concerning the mass spectrometer, the ionization energy of the ion source was set to 70 eV and the acquisition mode to 50–350 *m*/*z*. Compounds were identified through comparison of mass fragmentation spectra with reference NIST 98 spectra or by comparison of Kovats indexes and internal standard co-injection of pure standards (Sigma-Aldrich, Waltham, MA, USA).

#### 2.3.4. Moisture, Ashes, and Mineral Content

Water content was evaluated by drying the homogenized tomato fruits in an oven (Nuve, Ankara, Turkey) at 90 °C for at least 2 hours and until the dried weight remained constant. Through the weight loss, the percentage of water contained in the samples was calculated. The total mineral content was calculated by incubating the samples in a muffle at 400 °C for 24 h. For the determination of the mineral content, we digested tomato samples using a MARS 6 microwave digestion system (CEM, Matthews, NC, USA), as previously described [8]. Briefly, approximately 500 mg of dried tomato sample was weighed directly into a 100 mL polytetrafluoroethylene (PTFE) digestion vial, and then 9 mL of (65% *w*/*w*) HNO_3_ and 1 mL of (30% *w*/*v*) H_2_O_2_ were added. The temperature was gradually increased to 200 °C over 20 min and held for further 15 min to ensure complete digestion. Once cooled, the digested samples were diluted to a final volume of 50 mL with distilled H_2_O. All measurements were performed using a microwave plasma-atomic emission spectrometer (Agilent 4200 MP-AES) fitted with a double-pass cyclonic spray chamber and OneNeb nebulizer. N_2_ gas was supplied from a tank. All wavelengths were selected in the software library according to the sensitivity that was required. The calibration standards were prepared by diluting a 1000 mg/L multi-element standard solution (Sigma Aldrich, Waltham, MA, USA and Scharlab S.L., Barcelona, Spain) in 1% (*v*/*v*) HNO_3._

### 2.4. Ascorbic Acid Content

Ascorbic acid content (AAC) was determined as previously described by Bajaj [33], with some modifications. Briefly, the fresh fruits were crashed and homogenized, and then extracted 1:20 (*w*/*v*) using a mixture containing 0.05 M oxalic acid and 0.2 mM ethylenediaminetetraacetic acid (EDTA) for 10 min. Consequently, the extracts were filtered through a filter-paper and centrifuged (5000× *g*, 10 min, 4 °C). To 5 mL of the surfactant, we added 0.5 mL of a mixture containing 0.9 M HPO_3_ and 40% (*v*/*v*) acetic acid. Finally, 1 mL of 5% (*v*/*v*) H_2_SO_4_ and 2 mL of 5% (*w*/*v*) ammonium molybdate were added. After 15 min, the absorbance was measured at 760 nm against a blank prepared replacing the sample with 5 mL of the mixture of EDTA/oxalic acid. Quantification was performed using an external calibration curve of pure ascorbic acid (AA), and the results were expressed as milligram of AA per 100 g of FW.

### 2.5. Total Tocopherol Content

Total tocopherol content (TTC) was determined as previously reported [34]. Briefly, the fresh fruits were pulped and homogenized, and then placed in a Soxhlet apparatus with the aim of performing the extraction using hexane/ethyl acetate mixture at 85:15 (*v*/*v*). The extraction was performed in the dark for 8 h. Finally, samples were centrifuged (6000× *g*, 24 °C) for 10 min, and then 1 mL of the surfactant was added to 3 mL of a mixture containing 0.6 M H_2_SO_4_, 28 mM Na_2_PO_4_, and 4 mM MoNH_4_. After reduction of molybdenum during the incubation at 37 °C for 90 min, the absorbance of each sample was measured at 695 nm against a blank prepared replacing the sample with water. Quantification was performed using an external calibration curve of pure α-tocopherol, and the results were expressed as milligram of α-tocopherol per 100 g of FW.

### 2.6. Total Carotenoid Content

The total carotenoid content (TCC) was evaluated as previously reported [23], with some modifications [7]. Briefly, the homogenized tomato samples were extracted with 50:25:25 (*v*/*v*/*v*) hexane/acetone/ethanol mixture, using a 1:10 (*w*/*v*) ratio. After vortexing and centrifugation (10 min at 5000× *g*, 25 °C), the upper phase containing carotenoids was removed using a glass pipette and collected in a glass test tube. Extraction was repeated twice, and the different organic phases were pooled together. The combined organic fraction was firstly washed with 10 mL of saturated aqueous NaCl and then with 5 mL of 10% (*w*/*v*) K_2_CO_3_. Finally, the organic layer was dried with CaCl_2_. The excess of solvent was allowed to evaporate at room temperature for a few minutes in the dark. The entire extraction procedure was repeated twice with the aim to obtain 3 different technical replicates. One hundred milligrams of the obtained dried extract was mixed with 10 mL of 2:3 (*v*/*v*) acetone/hexane mixture for 1 min and then filtered on a paper filter. The absorbance was recorded at 3 different wavelengths (453, 505, and 663 nm) using a UV–VIS spectrophotometer (Cary 50, Agilent Technologies, Santa Clara, CA, USA), and TCC was calculated using the following formula:

$$TCC\;\left(\frac{mg}{100}mL\right)=0.216\;\times \;Abs_{663}-0.304\;\times \;Abs_{505}+0.452\;\times \;Abs_{453}$$

The obtained data were adjusted for both the extraction volume and sample weight, and expressed as micrograms of β-carotene per 100 g of FW.

### 2.7. Lycopene Content

The amount of lycopene was spectrophotometrically determined as previously described by Rayhan and colleagues [35], but with some modifications. Briefly, the fresh fruits were pulped and homogenized, and the obtained juices were extracted repeatedly using 1:1 (*v*/*v*) acetone/petroleum ether mixture until the complete color loss of the samples. Lycopene was collected in the petroleum ether fraction after the addition of an equal volume of deionized water. In order to completely remove lycopene from the acetone/water fraction, after the separation of the petroleum ether phase from the acetone/aqueous phase, we again extracted the acetone fraction with an equal amount of petroleum ether. The different petroleum ether fractions were combined, and the residual water content was removed by the adding Na_2_SO_4_ until the formation of a precipitate. The extraction procedure was repeated twice for each sample in order to obtain 3 different technical replicates. After an appropriate dilution, the color of the petroleum ether fraction was measured spectrophotometrically (Cary 50, Agilent Technologies, Santa Clara, CA, USA) in a quartz cuvette at 503 nm, against a blank consisting of petroleum ether. Lycopene content of the samples was calculated by using an external calibration curve of pure lycopene, and data were expressed as milligram of lycopene per 100 g of FW.

### 2.8. Total Polyphenol Content

Polyphenols were extracted from the fruits following the protocol previously described [34], with some modifications [36]. Briefly, the homogenized tomato samples were weighted and extracted with 90% (*v*/*v*) MetOH using a 1:10 (*w*/*v*) ratio. Samples were sonicated at room temperature for 15 min and then macerated under constant agitation for 24 h. After maceration, they were centrifuged (15 min at 6000× *g*, 4 °C) and the resulting supernatants were filtered. Methanolic extracts were stored at −20 °C until total polyphenol content (TPC) was determined via Folin–Ciocalteu assay, as previously reported [36]. Quantification was performed using an external calibration curve with gallic acid (GA). Analyses were performed in triplicate, and data were expressed as millimole GA equivalents (GAE) per 100 g of FW.

### 2.9. Evaluation of Antioxidant Properties

The same methanolic extracts used for TPC quantification were employed for the evaluation of the fruit antioxidant properties. The determination of the antioxidant properties included both the evaluation of radical scavenging activity via 2,2’-azino-bis(3-ethylbenzothiazoline-6-sulphonic acid (ABTS) and 2,2-diphenyl-1-picrylhydrazyl (DPPH) assay, and the evaluation of the reducing power via ferric reducing antioxidant power (FRAP) assay [34]. The inhibition percentage for each assay was measured using the following equation:

$$AA\%=\;\frac{A_{blank}-\;A_{sample}}{A_{blank}}\;\times \;100$$

where AA% is the percentage of color reduction of the reagent, A_blank_ is the absorbance of blank, and A_sample_ is the absorbance of the sample read at the specific wavelength of each assay. 6-Hydroxy-2,5,7,8-tetramethylchroman-2-carboxylic acid (Trolox) was employed as reference standard, and the antioxidant activity of each assay was expressed as millimole of Trolox equivalent (TE) per 100 g of FW.

#### 2.9.1. ABTS assay

In order to form the radical 2,2’-azino-bis(3-ethylbenzothiazoline-6-sulphonic acid (ABTS), we prepared 7 mM ABTS in distilled water and incubated it for 16 h with 2.45 mM K_2_S_2_O_8_ at room temperature. The radical ABTS^·+^ solution was diluted in methanol until reaching a final absorbance equal to 0.70 at 734 nm. For the assay, 1 mL of the ABTS mixture was incubated for 5 min with different dilutions of the previously prepared tomato methanolic extracts. The decay of the radical ABTS^·+^ resulting from the incubation with the different dilutions of tomato methanolic extracts was monitored by reading the color decrease at 734 nm.

#### 2.9.2. DPPH assay

At 1 mL of 0.1 mM 2,2-diphenyl-1-picrylhydrazyl (DPPH^·^), we added different concentrations of the previously prepared tomato methanolic extracts. After vigorous shaking, the mixture was incubated for 30 min in the dark and at 25 °C. The reduction of the radical DPPH resulting from the incubation with the different dilutions of tomato methanolic extracts was monitored by reading the color decrease at 517 nm.

#### 2.9.3. FRAP assay

To 300 mM CH_3_COONa, we added 36% (*v*/*v*) HCl until a pH value equal to 3.6 was measured. Consequently, 10 mM 2,4,6-Tris(2-pyridyl)-s-triazine (TPTZ) and 20 mM FeCl_3_ were added to the acetate mixture at an 8:1:1 (*v*/*v*/*v*) ratio. The composed mixture was then incubated at 37 °C and for 10 min with different concentrations of the previously prepared tomato methanolic extracts. The decay of the absorbance was monitored at 595 nm.

### 2.10. Statistycal Analysis

Differences in the pomological attributes at different harvest times were analyzed using the one-way analysis of variance (ANOVA; general linear model); meanwhile, those between the treatments at the same harvest time were tested with Tukey’s high significance difference (HSD) test at *p* ≤ 0.05. At least 5 different replicates were used for chemical analyses, and statistical differences were analyzed using the one-way analysis of variance (ANOVA; general linear model). All statistical analyses were performed using SPSS ver. 24 software (IMB, Armonk, NY, USA).

## 3. Results and Discussion

The improvement of the pomological traits is one of the main focuses of modern agriculture practices [37]. In particular, one of the main challenges is to find new methodologies that are able to reduce the fruit ripening time with the concomitant enhancement of pomological traits, such as fruit appearance, number, weight, and size [37]. However, in addition to the agronomic needs to maximize the production yield and to improve the pomological aspects of crop products, the consumers’ demand for products with high nutritional and nutraceutical values is also increasingly rising [22,38]. In this context, seaweed and yeast extracts are receiving a greater acceptance in horticulture as plant biostimulants, due to several beneficial effects against abiotic stresses [18,24,25,26]. However, the effects deriving from the application of biostimulants based on the combination of seaweed and yeast extracts on tomato fruit quality grown under standard conditions are scarcely available in the literature. For these reasons, in this work we evaluated whether the treatments with a commercial biostimulant based on seaweed and yeast extract could positively affect not only the agronomical aspects of tomato plants, but also the proximate composition, nutritional values, and nutraceutical properties of their fruits.

### 3.1. Biostimulant Treatments Reduced the Ripening Time and Increased Yield in Early Harvest Stages

Fruit maturation is one of the most important plant stages for crops, and it seriously affects the production times in agriculture. Among the possible strategies aimed to increase plant productivity, decreasing the fruit ripening times and increasing the production yield are the most popular solutions among farmers [17,37]. Consequently, “grow faster, grow more, but stronger” was for a long time the main objective of modern plant biotechnologies. As an alternative to plant engineering, specific products, including plant biostimulants, are widely used for this purpose [11,17,39]. Here, we evaluated the possible effect of the commercial plant biostimulant based on seaweed and yeast extract on tomato fruit maturation and yield.

**Figure 2:**
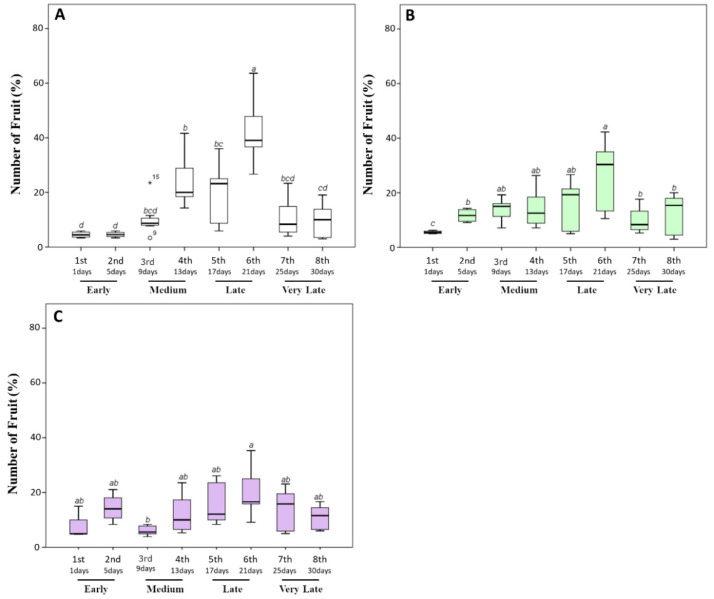
Fruit percentage (%) produced by tomato plants during the harvesting time (30 days). After the appearance of the first ripe fruits, the fully red-colored fruits were harvested every 4 days. (**A**) The percentage of fruit number harvested from untreated plants. (**B**,**C**) Those harvested from plants treated with a triple or a quadruple dosage of biostimulant, respectively. Within the same panel, the different lowercase letters on the top of each boxplot indicate significant differences at *p* ≤ 0.05, as measured by Tukey’s multiple range test. The letter “a” denotes the highest value. For additional statistical analysis information, see Table S1.

In our experimental conditions, both untreated and biostimulant-treated plants started to produce ripe fruits after about 75 days from transplanting. However, differences in the fruit number, weight, yield, and ripening time were observed within 30 days. In general, the untreated plants produced 22.08 ± 5.89 fruits per plant, of which about 80% during the medium/later harvest time (13–21 days after the appearance of the first ripe fruits). In particular, 17%, 21%, and 38% of the total fruit amount was collected between the fourth and sixth sampling times (Figure 2A).

On the other hand, tomato plants treated with a triple or quadruple dosage of biostimulant produced a non-significantly lower amount of fruit. In particular, 18.92 ± 3.63 and 19.83 ± 2.52 fruits were respectively produced by plants treated with the biostimulant three or four times. However, a statistically significant (*p* ≤ 0.05) decrease in the fruit percentage during the latest stages and an increase during the earliest harvest times was observed when tomato plants were treated with the biostimulant (Figure 2B,C). In particular, the percentage of the total fruit amount collected during the earliest stages increased from 8.18% ± 1.95% (untreated plants) to 17.21% ± 3.05% and to 21.75% ± 7.32% for plants treated three or four times, respectively. Consequently, the amount of fruit produced at the latest stages significantly (*p* ≤ 0.05) decreased from 42.51% ± 8.06% to 25.95% ± 6.74% and to 20.13% ± 8.12%, respectively. These data are indicative of a reduction in fruit ripening times after the application of the biostimulant. Our results are consistent with the findings of Soppelsa and colleagues, who treated strawberry plants with different products, including aminoacid-based biostimulant [12]. In particular, their results showed that, although the number of fruits did not significantly increase with the application of the biostimulant, the treated plants had an overall earlier ripening and a larger share of fruits picked during the earlier stages.

Finally, after the last harvesting time, the unripe fruits were also picked and counted. The untreated plants produced 8.35 ± 2.10 unripe fruits; meanwhile, 15.74 ± 3.59 and 17.23 ± 2.22 were harvested from plants treated with a triple or quadruple dosage of biostimulant, respectively. These data, although excluded from further analyses, suggested that plants treated with the biostimulant were still able to produce fruits compared to untreated plants.

**Figure 3:**
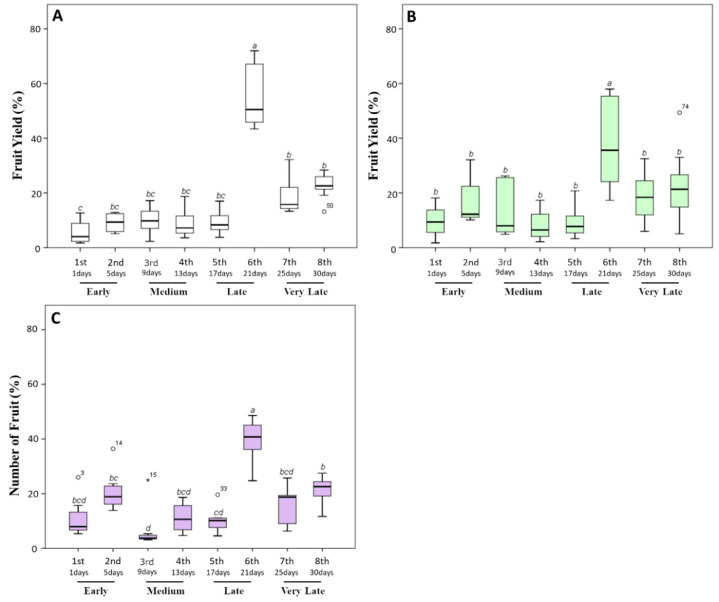
Fruit yield percentage (%) produced by tomato plants during the harvesting time (30 days). After the appearance of the first ripe fruits, the fully red-colored fruits were harvested every 4 days. (**A**) The fruit weight percentage harvested from untreated plants. (**B**,**C**) Those harvested from plants treated with a triple or a quadruple dosage of biostimulant, respectively. Within the same panel, the different lowercase letters on the top of each boxplot indicate significant differences at *p* ≤ 0.05, as measured by Tukey’s multiple range test. The letter “a” denotes the highest value. For additional statistical analysis information, see Table S1.

Concerning weight yield data, we recorded a production of 44.89 ± 6.62 g of tomato per plants in untreated plants, of which about 80% was harvested during the late and very late stages (sixth to eighth harvest times) (Figure 3A). In particular, 51%, 17%, and 21% of the total fruit production was collected starting from the 21st day. Similar data were also previously documented for untreated Micro-Tom grown in greenhouse in similar experimental conditions [40,41,42].

When tomato plants were treated with a triple (Figure 3B) or quadruple dosage (Figure 3C) of the biostimulant, a significant (*p* ≤ 0.05) decrease in the yield percentage during the late and very late stages and an increase during early times were observed. In particular, the yield percentage in the earliest stages increased from 8.56% ± 1.70% grams per plant (untreated plants) to 20.98% ± 4.04% (triple dosage of the biostimulant) and to 20.89% ± 3.38% (quadruple dosage of the biostimulant). Moreover, an increment in the total weight (from 44.89 ± 6.62 g per plant to 59.83 ± 5.58 and 54.50 ± 3.78 g, respectively, for triple and quadruple treated plants) was observed. The increase in yield production of Micro-Tom testing the action of biostimulants under greenhouse conditions has been reported in limited research studies [43]. Normally, the effects induced by the application of biostimulants on plant growth and crop productivity seem to be different from the nutritional effect observed after the application of commercial fertilizers. In particular, Colla et al. [44] and Ali et al. [45] showed that the improved productivity of tomato plants could be linked to signaling molecules, including polysaccharides, soluble peptides, oligopeptides, and free amino acids, which represent about the 30–40% of the total content of commercial seaweed extracts. Indeed, these molecules act in plants as signaling compounds regulating plant growth and development, and promoting endogenous phytohormonal biosynthesis [46].

### 3.2. Biostimulant Treatments Increased the Size of Tomato Fruits

Fruit size is an important factor directly correlated to the perception of high-quality fruits, and is of critical consideration not only from a consumer point of view, but also in terms of commercial value [7]. However, for most fruits produced by commercial cultivars, the size is too small to satisfy market demand. Consequently, farmers prefer to increase the fruit size at the expense of the total amount of fruits [47].

Micro-Tom is one of the crops that produces the smallest fruits within the genus *Solanum lycopersicum* [40]. Indeed, their external diameter ranges between a few hundred millimeters up to one and half centimeters, and only rarely does the fruit diameter exceed 1 centimeter [40]. For this reason, although the taste of Micro-Tom is completely comparable to other tomato varieties, this cultivar finds various difficulties in marketing due its bad production yield and fruit size; however, is largely employed as a laboratory model.

**Figure 4:**
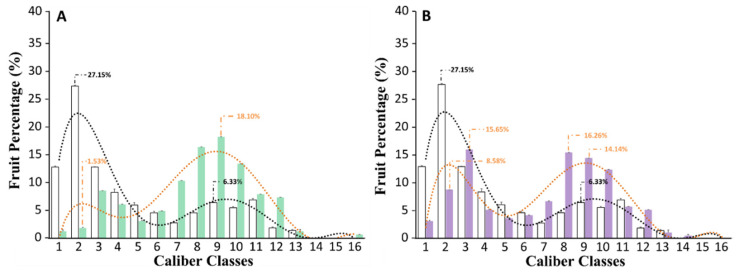
Size of the fruits produced by untreated tomato plants, or treated with a triple or quadruple dosage of biostimulant. (**A**) The fruit caliber of plants that were non-treated (grey) versus plants treated with a triple dosage (green) of biostimulant. (**B**) The fruit caliber of non-treated plants (grey) versus plants treated with a quadruple dosage (violet) of biostimulant. For each panel, the dotted line shows the distribution trend of fruit size (untreated = black; treated with the biostimulant = orange). The *x*-axis reports the caliber classes in which the fruits have been grouped according to their external diameter. The caliber classes and the external diameter of each group is reported in Table S2.

In this work, in order to understand if the biostimulant application could affect fruit size and uniformity, we classified the fully red-colored tomatoes harvested from untreated or biostimulant-treated plants on the basis of their external diameters into 16 different size classes (Figure 1, Table S2). Data concerning the classification and the dimension analysis are displayed in Figure 4.

In our experimental conditions, untreated plants produced very small fruits, and more than 50% of the total production had an external diameter (ED) ranging between 0.350 and 0.600 cm (Figure 4). In particular, 13% of the fruits belonged to the first caliber class (ED less than 0.450 cm), 27% to the second (ED comprising between 0.500 and 0.525 cm), and about 13% to the third (ED comprising between 0.525 and 0.600 cm). On the other hand, plants treated with a triple or quadruple dosage of the biostimulant significantly (*p* ≤ 0.05) produced fruits larger than untreated plants (Figure 4). Specifically, the best results were obtained when tomato plants were subjected to a triple dosage of the biostimulant treatment (Figure 4A). In this case, about 60% of the total production was concentrated between the 7th and 10th caliber classes (ED ranged between 0.825 and 1.125 cm). Similar results were also obtained from plants treated with a quadruple dosage of the biostimulant (Figure 4B). However, in this case, a large percentage of fruits with a lower ED was also counted.

Actually, the mechanism behind the increase of fruit yield and size after the treatment with the biostimulants is unclear and unknown. However, several authors correlated these effects to the potential intensification of plant enzymatic systems to the biostimulant chelating metal activity, or to their auxin- and gibberellin-like activity [12,19]. In particular, it was recently reported in the literature that the application of seaweed-based biostimulants on crop plants could lead not only to enhanced root development [27], biomass accumulation [19,26], and triggering in flowering and fruit set [48], but also to improved fruit yield and large sized fruits with superior quality [19,49].

### 3.3. Biostimulant Treatments Affected the Proximate Composition of Tomato Fruits

In general, biostimulants help to increase plant production by improving their fitness [17]. Their mode of action is not well understood yet, but several pieces of evidence support the beneficial effects of biostimulation also on food composition [15]. However, despite numerous studies focused their attention on plant growth and yield following biostimulant treatments, there are only few studies reporting the potential influence on the fruit quality [17,50]. These aspects are of special interest, particularly for those plants commonly used for the consumption or for the preparation of cosmetic, nutraceutical, and pharmaceutical products.

In our experimental conditions, both untreated and biostimulant-treated plants produced fruits with a moisture content of about 90%. Even if small significant (*p <* 0.05) differences were observed in the water content, important variations of the total amount of proteins, lipids, and sugars were recorded (Table 1). In particular, the most significant effect derived from the application of this biostimulant on tomato plants was observed on fruit lipid content and fatty acid profile. Indeed, tomato fruits harvested from plants treated with a triple dosage of the biostimulant had an enrichment of 20% in the total fat content, ranging from 0.528 ± 0.016 to 0.631 ± 0.018 mg per gram of FW. Accordingly with data previously reported in the literature [51], our GC–MS and GC–FID analyses allowed for the identification and the quantification of eight different fatty acids (Table 2). Unsaturated fatty acids (UFA) were the main fatty acids in our tomato samples, reaching about 75% of the total fatty acid content, both in untreated and biostimulant-treated samples. The main fatty acid detected in our samples was linoleic acid (C18:2ω6), followed by oleic acid (C18:1ω9), and palmitic acid (C16:0). However, after the treatment with the biostimulant, strong changes in their percentage content were observed. In particular, the percentage content of roughanic acid (C16:3ω3), palmitoleic acid (C16:1ϖ7), oleic acid (C18:1ϖ9), elaidic acid (C18:1ϖ9), and sapienic acid (C16:1ϖ-10) increased in parallel to the decrease of linoleic acid (C18:2ϖ6). On the other hand, saturated fatty acids did not vary significantly (Table 2).

Fatty acid profile is now a nutritional hot topic, and the presence of these molecules in foods is very attractive for the consumer due to the different benefits derived from their intake [31,32]. Among the variations in the fatty acid profile evaluated in our experiments, the increment of C16:3ω3 and the concomitant reduction in C18:2ω6 are the most interesting effects observed. Indeed, even if C16:3ω3 and C18:2ω6 are both essential fatty acids that cannot be synthesized in the human body and must therefore be absorbed through the diet, recent studies have shown that dietary imbalance of C18:2ω6/C16:3ω3 ratio can affect human health [52]. In particular, the concomitant intake of a large amount of ω6 and low amount of ω3 are associated to an increased production of pro-inflammatory cytokines such as tumor necrosis factor-alpha (TNF-α), interleukin-1 (IL-1), and interleukin-6 (IL-6), and thus excessively increase inflammation [52]. 

In the present study, we also evaluated the mineral composition and the content of some vitamins in tomato fruits harvested from both untreated and biostimulant-treated plants. This quantification is reported in Table 1. In our experimental conditions, independently from the treatments, K was the most abundant in all the analyzed fruits, ranging between 73 and 75% of the total mineral content; meanwhile, Na was only about 1.5%. When tomato plants were treated with the biostimulant, MP-AES analysis revealed changes in mineral profile (Table 1). In particular, the fruits harvested from biostimulant-treated plants were enriched in K, Na, P, Fe, Cu, Zn, Mn, and Mo, recording the highest values in plants treated with three biostimulant dosages.

Micronutrients are used in several plant physiological processes, and in a relatively small amount, constituting less than 0.1% of dry plant tissue [53]. The microelement uptake by plants depends both on the content originally present in the soil, as well as from the content that becomes available after plant watering or after plant treatment with chemicals, fertilizers, or biostimulants [54,55]. Plants increase micronutrient uptake in specific conditions, with the aim of increasing the binding of solar energy [55], improving the conversion of light into chemical energy [55], intensifying primary or secondary metabolism [54], or enhancing the enzymatic activity of a large number of enzymes [54]. Microelements are stored in different plant districts, including edible organs. The consumption of microelement-enriched fruits may have an important impact on human health due to their involvement in several biochemical processes. Indeed, insufficient dietary intakes of microelements impair several functions of the central nervous system, reproductive system, enzyme activities, and energy metabolism, thus leading to serious illnesses [53].

In our experimental conditions, we observed an average increase of about 15% of Fe, Cu, Zn, and Mn in tomato fruits produced by plants treated with the biostimulant (Table 1). The most significant effect was observed in tomato fruits harvested from plants treated with a triple dosage of the biostimulant. In this case, an increment of 24% and 21% was recorded for Zn and Cu, respectively.

Tomato fruits are also an excellent source of ascorbic acid, tocopherols, and retinol compared to other regularly consumed vegetables [56]. They are very important antioxidant vitamins that are involved in different human physiological pathways. For example, ascorbic acid is required for the correct functioning of several enzymes and contributes to the regular immune system function [57]. Tocopherols are a class of four different methylated phenols largely studied for their functional bioactivity, playing a key role in age-related macular degeneration and chronic diseases, such as cardiovascular disease, cancer, and Alzheimer’s disease. Finally, retinol is an essential vitamin resulting from the metabolism of β-carotene, whose deficiency results in serious illnesses [58].

Our spectrophotometric analyses were aimed at the direct quantification of the total content of ascorbic acid and tocopherols, and at the indirect estimation of retinol through the evaluation of the total carotenoid content (Table 1). Our data suggest that the application of the biostimulant did not drastically change the vitamin content, except for tocopherols. However, a strong limitation of spectrophotometric assays is the impossibility of quantifying each specific form of tocopherol or carotenoid. Consequently, we cannot exclude that the application of the biostimulant does not effectively affect the qualitative composition of carotenoids, even in cases in which quantitative changes were not recorded.

### 3.4. Biostimulant Treatments Did Not Affect the Phytochemical Composition of Tomato Fruits, but Increased Their Antioxidant Properties

Tomatoes are the second most important commercial crop worldwide, and they are a very important component of the Mediterranean diet [56,59]. The importance of tomato consumption is linked to its particular phytochemical composition as it is a rich source of bioactive and antioxidant compounds, including lycopene, carotenoids, and polyphenols [59].

Recent findings showed that biostimulant applications may be able to modify the plant primary and secondary metabolism, stimulating the production of bioactive compounds in roots, stems, leaves, and fruits [44,60]. For example, Kocira and colleagues, studying the effects resulting from the application of three different biostimulants from *Ulva fasciata*, *Sargassum ilicifolium*, and *Gracilaria corticata* on beans, reported not only an increase in yield, but also in the production of polyphenols and carotenoids [60]. Similar results were also described after the application of biostimulants on other plants, including *Prunus armeniaca* [14], *Phaseolus vulgaris* [61], *Malus pumila* [13], and *Solanum lycopersicum* [15].

**Figure 5:**
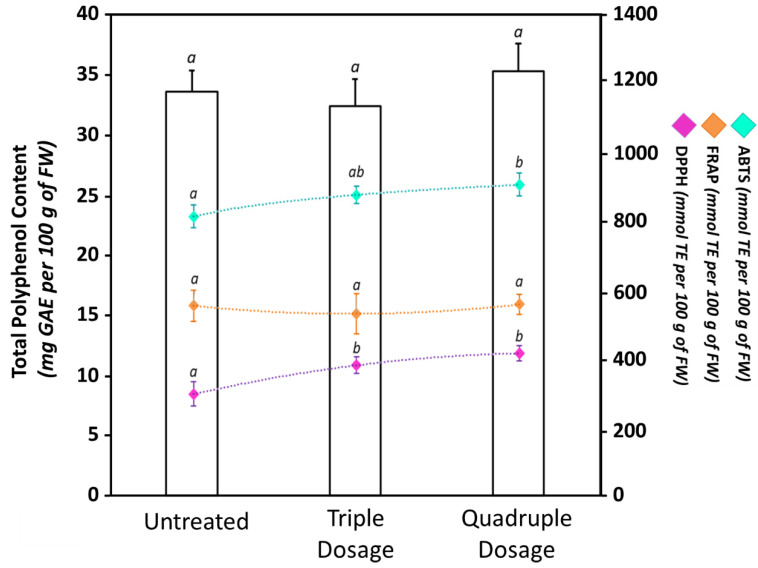
Total polyphenol content (TPC), radical scavenging activity (2,2’-azino-bis(3-ethylbenzothiazoline-6-sulphonic acid (ABTS) and 2,2-diphenyl-1-picrylhydrazyl (DPPH)), and ferric reducing antioxidant power (FRAP) of untreated and biostimulant-treated tomato fruits. The bars show the TPC measured by Folin–Ciocalteu assay; meanwhile, the points indicate the antioxidant properties measured by ABTS (light-blue), DPPH (violet), or FRAP (orange). Values are expressed as mean ± standard deviation (SD) of three experiments. Within the same dataset, different lowercase letters indicate significant differences at *p* ≤ 0.05 as measured by Tukey’s multiple range test. The letter “a” denotes the lowest content. For additional statistical information, see Table S6.

However, the treatment of crops with unconventional products may negatively affect the fruit quality by reducing their nutraceutical components [21,62,63,64]. For example, Grabowska et al. showed that treatments with commercial plant biostimulants on two different tomato cultivars increased the lycopene content in the fruits but also caused a strong reduction of other qualitative traits, including carotenes, ascorbic acid, soluble sugars, and total antioxidant activity [64]. Consequently, it is important not only for improving the agronomic aspects such as yield and size, but also to verify and guarantee that the fruit nutraceutical properties are not compromised. Consequently, another aim of our work was the evaluation of the total content of polyphenols (Figure 5), carotenoids, and lycopene (Table 1) both in untreated and biostimulant-treated fruits.

Plant polyphenols are highly desired components in the human diet due to their beneficial antioxidant properties and their preventive role in different oxidative stress-related illnesses, including cancer, cardiovascular diseases, and neurodegenerative diseases [38]. In our experimental conditions, TPC of Micro-Tom fruits ranged between 32.42 ± 2.27 mg GAE per 100 g of FW and 35.31 ± 2.32 mg GAE per 100 g of FW. Our determinations are in accordance with data reported in the literature for Micro-Tom by other authors [56,59]. When tomato plants were treated with the biostimulant, changes in TPC were not observed. While unusual, these results are closely comparable with data from other studies. For example, Stefania Sut and colleagues showed a positive impact of the application of six different biostimulants on growth and production of *Acmella oleracea* plants, with limited effects on the biosynthesis of secondary metabolites [65]. Despite TPC not being affected by the biostimulant treatments, variations in antioxidant properties were observed (Figure 5). In particular, we recorded an increase in the scavenging activity after the biostimulant application. Indeed, when tomato plants were treated with a triple dosage, DPPH and ABTS values were, respectively, 27% and 8% higher compared to untreated plants; meanwhile, they were 38% and 11% higher, respectively, in tomato plants treated four times. Changes in radical scavenging activity independently from TPC variations may be linked to specific qualitative changes in the polyphenol composition after the treatments [22] as well as to the presence of other non-polyphenolic antioxidant compounds, such as carotenoids, alkaloids, lycopene, and vitamins [38,66]. On the other hand, when the reducing activity was measured by FRAP assay, we did not register any statistically significant (*p* ≤ 0.05) difference between fruits harvested from untreated or biostimulant-treated plants. The antioxidant differences observed between ABTS/DPPH and FRAP assays may instead have been related to the variability in pH, hydrophilicity, or action mechanism of the three different assays [22,36,66].

Color changes in ripening tomato fruits are correlated to different events, including the transition of chloroplasts to chromoplasts, the degradation of chlorophyll, and the synthesis of carotenoids and lycopene [56]. Due to the consumer preference for fully colored fruits, researchers have assessed new methodologies to enhance their production [50]. In particular, it has been demonstrated that the application of fertilizers, chemicals, or biostimulants may affect their synthesis [25,49,50], following not only in early maturation, but also in bioactive component-enriched foods [15,50,67]. However, at the end of our experimentations, although the fruits harvested from biostimulant-treated plants gained a fully red color before the corresponding untreated plants, we did not observe significant (*p* ≤ 0.05) enrichment of the lycopene and β-carotene contents (Table 1). These data suggest that, although the biostimulant is capable of accelerating the normal maturation process promoting lycopene and carotenoid biosynthesis, it is not able to modify its typical phytochemical composition.

## 4. Conclusions

In the present study, we investigated the effect of the application of plant biostimulant based on seaweed and yeast extracts on tomato yield, maturation, and fruit quality. Our data show how the application of the biostimulant was able not only to drastically reduce fruit ripening times, but also to enhance the production yield in terms of both fruit amount and size, leading to more marketable products. Moreover, nutritional analysis revealed changes in proximate composition and mineral content, as well as increased level of unsatured fatty acids and micronutrients essential for human health. Finally, regarding the number of bioactive compounds, the obtained results indicate limited influence of the biostimulant treatments on the biosynthesis of secondary metabolites at the fruit level. Indeed, the total content of polyphenols, carotenoids, and lycopene were not significantly improved compared to fruits harvested from control plants. Despite this, an increase in antioxidant properties, in terms of radical scavenging activity, was observed. These data suggest that despite quantitative changes not occurring, possible and punctual changes in the qualitative composition of polyphenols and carotenoids may have occurred. In conclusion, the biostimulant application was able to reduce the ripening times and fruit size, while slightly increasing nutritional and nutraceutical values, leading to more marketable tomato fruits.

## Figures and Tables

**Table 1:** Nutritional, mineral, and bioactive compound composition of fruits harvested from untreated plants, or treated with a triple or quadruple dosage of the biostimulant. Data are expressed as mean ± SD. Within the same row, different lowercase letters indicate statistical differences among the samples, as measured by one-way ANOVA analysis followed by Tukey’s test (*p* ≤ 0.05). Letter “a” denotes the highest content. For additional statistical analysis information, see Table S4.

	Untreated	Triple Dosage	Quadruple Dosage
**Nutritional Content** *(g per 100 g of FW)*			
**Protein content**	14.569 ± 0.201 ^b^	15.038 ± 0.138 ^a^	14.712 ± 0.194 ^ab^
**Fat content**	0.528 ± 0.016 ^b^	0.631 ± 0.018 ^a^	0.531 ± 0.016 ^b^
**Sugar content**	2.285 ± 0.034 ^b^	2.411 ± 0.078 ^a^	2.364 ± 0.051 ^ab^
**TSS (°Brix)**	6.832 ± 0.321 ^a^	7.001 ± 0.175 ^a^	7.122 ± 0.489 ^a^
**Moisture content**	90.438 ± 0.154 ^a^	90.252 ± 0.143 ^a^	89.088 ± 0.015 ^b^
**Ashes**	0.761 ± 0.021 ^a^	0.783 ± 0.012 ^a^	0.758 ± 0.009 ^a^
**Available energy *(kcal)***	72.168 ± 1.084 ^c^	75.475 ± 1.026 ^a^	73.083 ± 1.124 ^b^
**Mineral Content** *(mg per 100 g of FW)*			
**K**	541.671 ± 0.772 ^ab^	549.724 ± 4.953 ^a^	529.188 ± 9.972 ^b^
**Na**	10.874 ± 0.283 ^c^	13.535 ± 0.471 ^a^	12.799 ± 0.271 ^b^
**Ca**	27.653 ± 0.469 ^a^	23.612 ± 0.425 ^b^	23.248 ± 0.139 ^b^
**Mg**	20.574 ± 0.053 ^a^	21.027 ± 0.375 ^a^	20.977 ± 0.424 ^a^
**P**	113.791 ± 2.546 ^b^	126.579 ± 2.487 ^a^	126.637 ± 0.996 ^a^
**Cl**	2.753 ± 0.033 ^b^	2.394 ± 0.080 ^c^	2.985 ± 0.047 ^a^
**Fe**	0.505 ± 0.022 ^b^	0.577 ± 0.014 ^a^	0.563 ± 0.02 ^a^
**Cu**	0.096 ± 0.003 ^b^	0.117 ± 0.008 ^a^	0.111 ± 0.007 ^a^
**Zn**	0.089 ± 0.005 ^c^	0.111 ± 0.006 ^a^	0.103 ± 0.002 ^b^
**Mn**	0.145 ± 0.002 ^b^	0.163 ± 0.004 ^a^	0.165 ± 0.006 ^a^
**Si**	0.506 ± 0.013 ^b^	0.625 ± 0.021 ^a^	0.386 ± 0.003 ^c^
**B**	0.046 ± 0.005 ^a^	0.052 ± 0.003 ^a^	0.049 ± 0.004 ^a^
**Mo**	0.013 ± 0.001 ^b^	0.016 ± 0.002 ^a^	0.014 ± 0.002 ^ab^
**Bioactive Compound Content** *(mg per 100 g of FW)*			
**Lycopene**	7.182 ± 0.173 ^a^	6.972 ± 0.312 ^a^	7.013 ± 0.392 ^a^
**Carotenoids**	13.524 ± 1.235 ^a^	14.091 ± 0.924 ^a^	13.892 ± 0.783 ^a^
**Tocopherols**	0.398 ± 0.057 ^b^	0.424 ± 0.036 ^ab^	0.445 ± 0.074 ^a^
**Ascorbic acid**	15.925 ± 1.267 ^a^	14.180 ± 2.202 ^a^	14.781 ± 1.892 ^a^
**Polyphenols**	35.652 ± 1.723 ^a^	33.420 ± 2.279 ^a^	35.318 ± 2.323 ^a^

**Table 2:** Fatty acid percentage composition of untreated and biostimulant-treated tomato fruits. Data are expressed as mean ± SD. Within the same row, different lowercase letters indicate statistical differences among the samples, as measured by one-way ANOVA analysis followed by Tukey’s test (*p* ≤ 0.05). Letter “a” denotes the highest content. For additional statistical analysis information, see Table S5.

	Untreated	Triple Dosage	Quadruple Dosage
**C16:3ω3**	0.202 ± 0.026 ^b^	0.866 ± 0.009 ^a^	0.236 ± 0.007 ^b^
**C16:1ω7**	0.431 ± 0.044 ^b^	0.655 ± 0.102 ^a^	0.404 ± 0.015 ^b^
**C16:1ω10**	0.089 ± 0.017 ^a^	0.155 ± 0.012 ^a^	0.079 ± 0.054 ^a^
**C16:0**	19.734 ± 0.935 ^a^	19.604 ± 0.150 ^a^	19.997 ± 1.925 ^a^
**C18:2ω6**	48.984 ± 2.988 ^a^	37.964 ± 0.013 ^b^	49.220 ± 3.007 ^a^
**C18:1ω9**	24.441 ± 1.262 ^a^	27.154 ± 2.273 ^a^	23.848 ± 0.359 ^a^
**C18:1ω9**	1.410 ± 0.122 ^b^	2.352 ± 0.039 ^a^	1.332 ± 0.045 ^b^
**C18:0**	4.128 ± 0.181 ^a^	4.060 ± 0.045 ^a^	4.516 ± 0.336 ^a^
**SFA**	23.863 ± 1.117 ^b^	29.664 ± 0.195 ^a^	24.514 ± 2.261 ^b^
**UFA**	75.559 ± 4.459 ^a^	69.149 ± 2.449 ^a^	75.122 ± 3.490 ^a^
**MUFA**	26.372 ± 1.445 ^b^	30.318 ± 2.427 ^a^	25.664 ± 0.475 ^b^
**PUFA**	49.187 ± 3.014 ^a^	38.831 ± 0.022 ^b^	49.457 ± 3.015 ^a^
**SFA/UFA**	0.315 ± 0.013 ^b^	0.428 ± 0.014 ^a^	0.326 ± 0.009 ^b^

## Supplementary Materials

The following are available online at https://www.mdpi.com/2218-273X/10/12/1662/s1: Table S1: Tukey’s high significance difference (HSD) post hoc differences in fruit number percentage and fruit weight percentage produced during the experimentation time (30 days) and collected during the specific harvest time. Table S2: Percentage of fruits harvested according to their external diameter. Table S3: Tukey’s HSD post hoc differences in fruit size of ripe fruits produced during the experimentation time (30 days) and collected during the specific harvest time. Table S4: Tukey’s HSD post hoc differences in proximate composition, mineral content, and bioactive compounds determined in untreated and biostimulant-treated tomato fruits. Table S5: Tukey’s HSD post hoc differences in fatty acid composition. Table S6: Tukey’s HSD post hoc differences in radical scavenging activity (DPPH and ABTS), reducing activity (FRAP), and total polyphenol content (TPC).
