# Supplementary material for: The Application of a Plant Biostimulant Based on Seaweed and Yeast Extract Improved Tomato Fruit Development and Quality
Source: Biomolecules. 2020 Dec 12;10(12):1662. doi: 10.3390/biom10121662 (PMC7763504; doi:10.3390/biom10121662)
Supplement: Supplementary file 1 [file biomolecules-10-01662-s001.zip › STable1.docx]

**Supporting Table 1**: Tukey’s HSD post hoc differences in Fruit Number Percentage and Fruit Weight Percentage produced during the experimentation time (30 days) and collected during the specific Harvest Time. *P<0.05; **P<0.01; ***P<0.005.

| **Harvest Time** | | **HSD post-hoc** | | | | | |
| --- | --- | --- | --- | --- | --- | --- | --- |
|  |  | **Number of Fruits** | | | **Weight of Fruits** | | |
|  |  | **Control** | **Treatment I** | **Treatment II** | **Control** | **Treatment I** | **Treatment II** |
| **1st** | **2nd** | 0.05 | 6.13 | 6.95 | 3.55 | 7.41 | 9.68 |
|  | **3rd** | 5.85 | 8.17 | 1.24 | 4.31 | 3.32 | 4.4 |
|  | **4th** | 19.71*** | 8.65 | 4.59 | 3.06 | 1.71 | 0.13 |
|  | **5th** | 14.56 | 10.14 | 7.9 | 3.79 | 0.37 | 1.06 |
|  | **6th** | 37.98*** | 20.41*** | 12.73* | 49.68*** | 29.04*** | 28.17*** |
|  | **7th** | 6.33 | 4.5 | 6.69 | 14.03* | 5.11 | 4.06 |
|  | **8th** | 5 | 6.35 | 3.47 | 15.21 | 11.39 | 10.81* |
| **2nd** | **3rd** | 5.79 | 2.04 | 8.19 | 0.75 | 4.07 | 14.09** |
|  | **4th** | 19.66*** | 2.52 | 2.35 | 0.49 | 9.11 | 9.82 |
|  | **5th** | 14.5 | 4 | 0.94 | 0.23 | 7.77 | 10.74 |
|  | **6th** | 37.93*** | 14.28* | 5.78 | 46.13*** | 21.64*** | 18.48*** |
|  | **7th** | 6.28 | 1.63 | 0.26 | 10.48 | 2.28 | 5.62 |
|  | **8th** | 4.94 | 0.21 | 3.48 | 11.65 | 3.99 | 1.12 |
| **3rd** | **4th** | 13.86* | 0.48 | 5.83 | 1.24 | 5.04 | 4.27 |
|  | **5th** | 8.7 | 1.96 | 9.14 | 0.51 | 3.71 | 3.34 |
|  | **6th** | 32.13*** | 12.23* | 13.97*** | 45.37*** | 25.71*** | 32.58*** |
|  | **7th** | 0.48 | 3.67 | 7.93 | 9.72 | 1.78 | 8.47 |
|  | **8th** | 0.85 | 1.82 | 4.71 | 10.89 | 8.06 | 15.22*** |
| **4th** | **5th** | 5.15 | 1.48 | 3.3 | 0.72 | 1.33 | 0.92 |
|  | **6th** | 18.27*** | 11.75** | 8.14 | 46.62*** | 30.75*** | 28.3*** |
|  | **7th** | 13.38** | 4.15 | 2.09 | 10.97* | 6.82 | 4.19 |
|  | **8th** | 14.71* | 2.3 | 1.12 | 12.14 | 13.11 | 10.94* |
| **5th** | **6th** | 23.42*** | 10.27 | 4.83 | 45.89*** | 29.42*** | 29.23*** |
|  | **7th** | 8.22 | 5.64 | 1.21 | 10.24 | 5.48 | 5.12 |
|  | **8th** | 9.55 | 3.78 | 4.43 | 11.41 | 11.77 | 11.87* |
| **6th** | **7th** | 31.65*** | 15.91*** | 6.04 | 35.64*** | 23.93*** | 24.11*** |
|  | **8th** | 32.98*** | 14.06** | 9.26 | 34.47*** | 17.64** | 17.36*** |
| **7th** | **8th** | 1.33 | 1.85 | 3.22 | 1.16 | 6.28 | 6.74 |
