# Supplementary material for: The Application of a Plant Biostimulant Based on Seaweed and Yeast Extract Improved Tomato Fruit Development and Quality
Source: Biomolecules. 2020 Dec 12;10(12):1662. doi: 10.3390/biom10121662 (PMC7763504; doi:10.3390/biom10121662)
Supplement: Supplementary file 1 [file biomolecules-10-01662-s001.zip › STable2.docx]

**Supporting Table 2**: Percentage of fruits harvested according to their external diameter. After the collection of the ripe fruits during the experimentation time (30 days), the fruits were divided in sixteen different classes according to their external diameters. Within the same row, different lowercase letters indicate significant differences at *p* ≤ 0.05 between untreated plants (control) and biostimulant-treated plants, as measured by Tukey’s multiple range test. Letter “a” denotes the highest content. For further statistical information see Table S3.

| **Caliber**  **Class** | **Diameter**  **(cm)** | **Fruit Percentage (%)** | | |
| --- | --- | --- | --- | --- |
|  |  | **Control** | **Treatment 1** | **Treatment 2** |
| **1** | *< 0.450* | 12.66 ± 2.30^a^ | 1.20 ± 0.25^b^ | 3.03 ± 0.22^b^ |
| **2** | *0.450 - 0.525* | 27.14 ± 4.62^a^ | 1.83 ± 0.94^b^ | 8.58 ± 1.12^b^ |
| **3** | *0.525 - 0.600* | 12.66 ± 0.52^a^ | 8.43 ± 2.72^a^ | 15.65 ± 4.74^a^ |
| **4** | *0.600 - 0.675* | 8.14 ± 5.01^a^ | 6.02 ± 3.02^a^ | 5.05 ± 4.55^a^ |
| **5** | *0.675 - 0.750* | 5.88 ± 2.72^a^ | 3.01 ± 0.61^a^ | 3.53 ± 1.90^a^ |
| **6** | *0.750 - 0.825* | 4.52 ± 1.11^a^ | 4.81 ± 0.63^a^ | 4.04 ± 2.36^a^ |
| **7** | *0.825 - 0.900* | 2.71 ± 0.50^a^ | 10.24 ± 3.41^b^ | 6.56 ± 1.84^ab^ |
| **8** | *0.900 - 0.975* | 4.52 ± 1.32^a^ | 16.26 ± 4.92^b^ | 15.15 ± 0.78^b^ |
| **9** | *0.975 - 1.050* | 6.33 ± 0.74^a^ | 18.07 ± 3.03^b^ | 14.14 ± 2.02^b^ |
| **10** | *1.050 - 1.125* | 5.42 ± 0.91^b^ | 13.25 ± 3.22^a^ | 12.12 ± 4.73^a^ |
| **11** | *1.125 - 1.200* | 6.78 ± 1.82^a^ | 7.83 ± 1.91^a^ | 5.55 ± 4.11^a^ |
| **12** | *1.200 - 1.275* | 1.80 ± 0.32^b^ | 7.22 ± 3.52^a^ | 5.05 ± 1.52^b^ |
| **13** | *1.275 - 1.350* | 1.35 ± 0.24^a^ | 1.21 ± 0.32^a^ | 1.01 ± 0.44^a^ |
| **14** | *1.350 - 1.425* |  |  | 0.52 ± 0.10 |
| **15** | *1.425 - 1.500* |  |  |  |
| **16** | *>1.575* |  | 0.60 ± 0.01 |  |
