# Supplementary material for: The Application of a Plant Biostimulant Based on Seaweed and Yeast Extract Improved Tomato Fruit Development and Quality
Source: Biomolecules. 2020 Dec 12;10(12):1662. doi: 10.3390/biom10121662 (PMC7763504; doi:10.3390/biom10121662)
Supplement: Supplementary file 1 [file biomolecules-10-01662-s001.zip › STable4.docx]

**Supporting Table 4**: Tukey’s HSD post hoc differences in proximate composition, mineral content, and bioactive compounds determined in untreated and biostimulant treated tomato fruits. *P<0.05; **P<0.01; ***P<0.005.
