# Supplementary material for: The Application of a Plant Biostimulant Based on Seaweed and Yeast Extract Improved Tomato Fruit Development and Quality
Source: Biomolecules. 2020 Dec 12;10(12):1662. doi: 10.3390/biom10121662 (PMC7763504; doi:10.3390/biom10121662)
Supplement: Supplementary file 1 [file biomolecules-10-01662-s001.zip › STable6.docx]

**Supporting Table 5**: Tukey’s HSD post hoc differences in radical scavenging activity (DPPH and ABTS), reducing activity (FRAP), and TPC. *P<0.05; **P<0.01; ***P<0.005.

| **HSD Post hoc differences** | | | | | |
| --- | --- | --- | --- | --- | --- |
|  |  | **DPPH** | **ABTS** | **FRAP** | **TPC** |
| **Untreated** | **Single-Dosage** | -82.48* | -61.54 | 22.97 | 1.23 |
|  | **Double-Dosage** | -116.78** | -90.89* | -3.88 | -1.66 |
| **Single Dosage** | **Untreated** | 82.48* | 61.54 | -22.97 | -1.23 |
|  | **Double-Dosage** | -34.3 | -29.35 | -26.85 | -2.89 |
| **Double Dosage** | **Untreated** | 116.78** | 90.89* | 3.88 | 1.66 |
|  | **Single-Dosage** | 34.3 | 29.35 | 26.85 | 2.89 |
